# Supplementary material for: BAOS-CNN: A novel deep neuroevolution algorithm for multispecies seagrass detection
Source: PLoS One. 2024 Jun 25;19(6):e0281568. doi: 10.1371/journal.pone.0281568 (PMC11198790; doi:10.1371/journal.pone.0281568)
Supplement: Attachment — Submitted filename: Response_reviewers.docx [file pone.0281568.s001.docx]

**Manuscript** Number: PONE-D-23-00889

**Original Article Title: “**BAOS-CNN: A novel deep neuroevolution algorithm for multispecies seagrass detection”

**Journal Requirements:**

| **Comment#1** | 1. Please ensure that your manuscript meets PLOS ONE's style requirements, including those for file naming. The PLOS ONE style templates can be found at    https://journals.plos.org/plosone/s/file?id=wjVg/PLOSOne_formatting_sample_main_bod y.pdf and https://journals.plos.org/plosone/s/file?id=ba62/PLOSOne_formatting_sample_title_autho rs_affiliations.pdf |
| --- | --- |
| **Author response:** | Thank you so much for this comment. We are sharing editable word format. Happy to change any other formats. |

| **Comment#2** | 2. Please note that PLOS ONE has specific guidelines on code sharing for submissions in which author-generated code underpins the findings in the manuscript. In these cases, all author-generated code must be made available without restrictions upon publication of the work.  Please review our guidelines at https://journals.plos.org/plosone/s/materials-and-softwaresharing#loc-sharing-code and ensure that your code is shared in a way that follows best practice and facilitates reproducibility and reuse. |
| --- | --- |
| **Author response:** | Thank you so much. The source code will be made publicly available using GitHub upon acceptance of the paper. Here is the GitHub link:  https://github.com/Kislunoman/BAOS-CNN |
| **Comment#3** | 3. We note that the grant information you provided in the ‘Funding Information’ and ‘Financial Disclosure’ sections do not match.   When you resubmit, please ensure that you provide the correct grant numbers for the awards you received for your study in the ‘Funding Information’ section.     4. Thank you for stating the following in the Acknowledgments Section of your manuscript:     "This research work is funded by the Australian Government Research Training Program (RTP) scholarship and supported by School of Science and Centre for Marine Ecosystems Research, Edith Cowan University, Australia.."    We note that you have provided funding information that is not currently declared in your  Funding Statement. However, funding information should not appear in the Acknowledgments section or other areas of your manuscript. We will only publish funding information present in the Funding Statement section of the online submission form.    Please remove any funding-related text from the manuscript and let us know how you would like to update your Funding Statement. Currently, your Funding Statement reads as follows:    "NO - Include this sentence at the end of your statement: The funders had no role in study design, data collection and analysis, decision to publish, or preparation of the manuscript."    Please include your amended statements within your cover letter; we will change the online submission form on your behalf. |
| **Author response:** | We would like to thank the academic editor for notifying the discrepancy. The funding statement and the acknowledgement section are now modified as follows:    **Funding Statement:** This research work is supported by the Australian Government Research Training Program (RTP) scholarship and the School of Science and Centre for Marine Ecosystems Research, Edith Cowan University, Australia.    **Acknowledgement:**  The authors would like to thank Kieryn Kilminster, Department of Water and Environmental Regulation (DWER) and Department of Biodiversity, Conservation and Attractions for their providing some of the seagrass images used in this research. |
| **Comment#4** | In your Data Availability statement, you have not specified where the minimal data set underlying the results described in your manuscript can be found. PLOS defines a study's minimal data set as the underlying data used to reach the conclusions drawn in the manuscript and any additional data required to replicate the reported study findings in their entirety. All PLOS journals require that the minimal data set be made fully available. For more information about our data policy, please see http://journals.plos.org/plosone/s/dataavailability.    Upon re-submitting your revised manuscript, please upload your study’s minimal underlying data set as either Supporting Information files or to a stable, public repository |
|  | and include the relevant URLs, DOIs, or accession numbers within your revised cover letter. For a list of acceptable repositories, please see http://journals.plos.org/plosone/s/dataavailability#loc-recommended-repositories. Any potentially identifying patient information must be fully anonymized.    Important: If there are ethical or legal restrictions to sharing your data publicly, please explain these restrictions in detail. Please see our guidelines for more information on what we consider unacceptable restrictions to publicly sharing data:  http://journals.plos.org/plosone/s/data-availability#loc-unacceptable-data-accessrestrictions. Note that it is not acceptable for the authors to be the sole named individuals responsible for ensuring data access.    We will update your Data Availability statement to reflect the information you provide in your cover letter.    We note that you have stated that you will provide repository information for your data at acceptance. Should your manuscript be accepted for publication, we will hold it until you provide the relevant accession numbers or DOIs necessary to access your data. If you wish to make changes to your Data Availability statement, please describe these changes in your cover letter and we will update your Data Availability statement to reflect the information you provide. |
| **Author response:** | The seagrass dataset used in this research comprised a total of 5,201 images obtained from various sources, including the Centre for Marine Ecosystems Research at Edith Cowan University, Western Australia, the Department of Water and Environmental Regulation, Western Australia, and the Department of Biodiversity, Conservation and Attractions (DBCA), Australia. We are currently in the process of obtaining approval from these organizations to publicly release the dataset. Once the necessary approvals have been obtained, we will make the dataset publicly available for use. |
| **Comment#5** | Please upload a new copy of Figure 5 as the detail is not clear. Please follow the link for more information: https://blogs.plos.org/plos/2019/06/looking-good-tips-for-creating-your-plos-figuresgraphics/  [https://blogs.plos.org/plos/2019/06/looking-good-tips-for-creating-your-plos-figuresgraphics/](https://blogs.plos.org/plos/2019/06/looking-good-tips-for-creating-your-plos-figures-graphics/) |
| **Author response:** | Thank you so much for your valuable comment. We have changed the Figure 5. |
| **Comment#6** | Please review your reference list to ensure that it is complete and correct. If you have cited papers that have been retracted, please include the rationale for doing so in the manuscript text, or remove these references and replace them with relevant current references. Any changes to the reference list should be mentioned in the rebuttal letter that accompanies your revised manuscript. If you need to cite a retracted article, indicate the article’s retracted status in the References list and also include a citation and full reference for the retraction notice. |
| **Author response:** | Thank you so much for this concern. We have checked all the references. |

We are pleased to see that reviewers have found the manuscript to be technically sound, supported by appropriate data, and the experiments conducted rigorously. They also confirmed that the statistical analysis has been performed appropriately and rigorously further validates the findings. We also appreciate the reviewers’ acknowledgment that the manuscript is presented in an intelligible fashion and written in standard English.

|  | **Reviewer 1** |  |  |
| --- | --- | --- | --- |
| **Comment 1:** | |  | The paper presents Deep Neuroevolutionary (DNE) model that can automate the architectural engineering. Since the authors are proposing novel deep neuroevolution algorithm, I am not able to find related work in the paper that can justify this novel contribution.  Author must add related work and compare their novel deep neuroevolution algorithm with other similar or near-to-similar proposed algorithms. |
| **Author response:** | |  | Thank you for your valuable and insightful feedback. We have carefully considered your comments and have made the necessary revisions to address your concerns. In the revised version of the paper, we have included a comprehensive review of relevant literature and compared our algorithm with other similar or near-to-similar proposed algorithms. This addition will provide a better understanding of the novelty and uniqueness of our approach.    **Added section (in Introduction):** Deep Neuroevolutionary algorithms combine the power of deep neural networks (which excel at learning complex patterns) and representations, with neuroevolutionary algorithms that optimize neural network architectures and parameters using evolutionary principles. Ijjina et al. [35] proposed a neural network classifier that utilized the power of a genetic algorithm to optimize the initial weights. Their approach mitigated the challenges of overfitting and local minimum issues. They achieved an accuracy of 99.98% in human action recognition on the UCF50 dataset. Martin et al. [36] proposed the EvoDeep algorithm, a deep neuroevolutionary algorithm to optimize both the parameters and architecture of Deep Neural Networks (DNNs). Their approach resulted in an accuracy of 98.93% on the handwritten digit dataset. Sun et al. [37] proposed the EvoCNN algorithm using genetic algorithms for evolving the architectures and connection weight initialization values of a deep convolutional neural network to address image classification problems. Davoudi et al. (2021) proposed a deep neuroevolutionary algorithm powered by genetic algorithms (GA) for the classification of Breast Cancer images. Their method outperformed the traditional Adam optimizer, achieving an image classification accuracy of 85%. Sajad et al. [38] proposed an enhanced variant of the Boosted Salp Swarm Algorithm (BSSA) to improve the original version of the Salp Swarm Algorithm (SSA). This enhancement involved the integration of two powerful optimization operators: opposition-based learning (OBL) and chaotic maps. Their two-stage deep neuroevolution (DNE) framework was developed to enhance the classification of chest X-ray images. By applying deep neuroevolutionary techniques, researchers aim to enhance the performance, efficiency, and adaptability of deep learning models. Jalali et al. [39] proposed an evolutionary algorithm by improving the basic version of competitive swarm optimizer by using three powerful evolutionary operators: Cauchy Mutation (CM), Evolutionary Boundary Constraint Handling (EBCH), and tent chaotic map. Their deep neuroevolutionary algorithm automated the identification process of COVID-19 disease from chest X-ray images, achieving an average accuracy of 98.56%. By reviewing the literature, we can see a significant advancement and immense potential in developing and hyper-tuning image classification models. |

| **Comment 2:** | How the evaluation of the fitness value of the candidates are computed. |
| --- | --- |
| **Author response:** | In the revised manuscript, we have provided a more explicit explanation of the fitness evaluation process and have included a comprehensive flow chart with a step-by-step example. We believe these additions will enhance the readers' understanding of our algorithm.    **Added section (in Section 1.5):**    To compute and optimize the fitness value for updating the hyperparameters of a CNN using BAOS algorithm, the following five basic steps are followed:     1. **Initialize Hyperparameters**: The first step is to initialize the electrons, which represent the different sets of parameters for the CNN. Each electron is randomly assigned an energy level and an orbital position. These hyperparameters will be part of the solution space that the evolutionary algorithm will explore.      2. **Encoding:** Encode the hyperparameters into a suitable representation that the BAOS Algorithm can work with. The proposed BAOS helps to determine the binding energy (BE) and the binding state (BS) of the atom and finds out the candidate with the lowest energy level (LE). Each solution represents one of the nine hyperparameters with a ninedimensional vector $X-{ij}, i=1,2,…, n$ and each hyperparameter is   $j=1,2,…,9$.     3. **Solution Generation:** Generate initial solutions by randomly selecting or generating hyperparameter configurations within the defined solution space. Each solution corresponds to a specific set of hyperparameters for the CNN.      4. **Solution Update:** The fourth step is to update the electrons' energy levels and positions with Lévy distribution based on their current positions and the positions of their neighbouring electrons. The electrons' positions are updated using a quantum-inspired mechanism that allows them to jump to different energy levels and explore the search space.      5. **Fitness Evaluation:** This step involves the performance evaluation of the CNN based on the electron's position in the orbital space. This can be done by training the CNN with the corresponding set of parameters and measuring its performance using performance metrics such as accuracy or mean squared error. During training, the hyperparameters are used to construct and configure the CNN model. The training process involves feeding the dataset through the CNN, and updating the network's hyperparameters and weights. The final step involves assessing the performance of the CNN on the specified evaluation metrics.      6. **Fitness Calculation:** Assign a fitness value to each solution based on the CNN's performance on the evaluation metrics. Higher fitness values are assigned to solutions that result in better performance. The fitness value can be determined directly from the evaluation metrics. The evolutionary algorithm can explore and identify hyperparameter settings during the training time on the dataset.      7. **Selection:** The best set of parameters is selected based on the corresponding electrons with the lowest energy levels. |

|  | 8. **Repeat:** By iteratively applying these steps within the BAOS algorithm, the hyperparameters of the CNN are optimized and updated based on their fitness values. This process allows for the identification of hyperparameter configurations that improve the CNN's performance on the chosen evaluation metrics. The process of evaluation, update, and selection is repeated until the optimal solution is found. |
| --- | --- |
| **Comment 3:** | Kindly explain flow chart with the help of example. There is no appropriate explanation of algorithm 1 in the paper. |
| **Author response:** | In the revised manuscript, we have revised and expanded the explanation of Algorithm 1 to provide a more accurate and detailed description. We believe this revision will provide clarity and assist readers in understanding the algorithmic steps.    **Added section (in Section 1.5):** The proposed BAOS-CNN algorithm combines the Atomic Orbital Search (AOS) algorithm with the Lévy flight technique to optimize the hyperparameters of convolutional neural networks (CNNs). The algorithm starts by initializing a population of solution candidates representing different hyperparameter configurations for the CNNs. Fitness values are evaluated, and binding states and binding energies are determined based on these fitness values. The algorithm then applies a Probability Density Function to identify promising solutions. Random parameters are updated, and the Photon Rate (PR) is calculated and adjusted using the Lévy distribution. The algorithm iterates through the population, updating parameters, and positions, and checking boundaries. This algorithm follows an iterative process to search for optimal hyperparameters. The algorithm initializes a population of solution candidates and evaluates their fitness values, identifying binding states and energies. Through the iteration loop, the algorithm updates parameters and positions based on specific rules and calculates the Photon Rate (PR) using the Lévy distribution. Repair and boundary checks are conducted to ensure valid solutions. The best solution, as determined by the Lévy distribution, is then used to adjust the CNNs model. Finally, the adjusted CNNs model is applied to classify images in the test set, completing the algorithm's optimization process for hyperparameter selection in CNNs. |
| **Comment 4:** | Is ECU-MSS-2 confidential dataset? |
|  | The "ECU-MSS-2" dataset comprises a total of 5,201 images obtained from various sources, including the Centre for Marine Ecosystems Research at Edith Cowan University, Western Australia, the Department of Water and Environmental Regulation, Western Australia, and the Department of Biodiversity, Conservation and Attractions (DBCA), Australia. We are currently in the process of obtaining approval from these organizations to publicly release the dataset. Once the necessary approvals have been obtained, we will make the dataset publicly available for use. |
| **Comment 5:** | Present the comparison between qualitative and quantitative evaluation in tabular format |
| **Author response:** | We have carefully considered your suggestion to present a comparison between qualitative and quantitative evaluation in a tabular format. In the Quantitative Evaluation section, we compared the performance of our models with six wellknown evolutionary algorithm-based CNN models. Additionally, we conducted a comparison using both four and five classes publicly available datasets. On the |
|  | other hand, the qualitative evaluation section presented a visual analysis, showcasing the performance of our models on various images. It is important to note that these two analyses are distinct in nature and not intended for direct comparison with each other. |

|  | Reviewer 2 |  | The paper presents a significant advancement in the field of metaheuristic algorithms, by combining the Lévy flight technique with the recently proposed Atomic Orbital Search (BAOS) algorithm. The innovative approach and promising results of the BAOS-CNN framework, particularly in automating architectural engineering and hyperparameter tuning of CNNs, is commendable.    The highlight of the paper is undoubtedly the application of BAOS-CNN to optimize a CNN model on a multi-species seagrass dataset. The achieved accuracy of 97.48%, which outperforms seven other evolutionary algorithms, is impressive. The use of BAOS to create a random population and evolve a new generation for searching the optimal architecture and hyperparameters combination of the CNN models showcases the power and versatility of this novel algorithm.    The authors' success in achieving high accuracy on different versions of the publicly available 'DeepSeagrass' dataset further underscores the effectiveness of the proposed BAOS-CNN model. The fact that it outperforms previously reported state-of-the-art accuracies on this dataset is a substantial accomplishment.    The paper also provides a valuable contribution by minimizing the requirement of manual CNN model architectural engineering and hyperparameter tuning. This feature enhances the practicality and usability of the algorithm in real-world applications, making it a potentially powerful tool in image classification or object detection in marine or other submerged environments.    In terms of future work, the authors' plan to apply ensemble learning techniques to enhance the performance by combining multiple evolutionary algorithm-based CNNs’ performances is promising. This approach could further improve the accuracy of the model and broaden the range of its applications.    Overall, the authors should be commended for their innovative work in developing and applying the BAOS-CNN framework. The paper is well-structured, and the findings are clearly presented. The proposed algorithm has great potential and sets the stage for exciting future developments in the field of deep neuroevolutionary algorithms. I look forward to seeing these future developments and the potential real-world applications that this algorithm could enable. |
| --- | --- | --- | --- |
|  |  |  |  |
| **Author**  **Response:** | |  | We are delighted that you found our work to be a significant advancement in the field of metaheuristic algorithms. Your insightful comments and positive feedback have truly encouraged and motivated us. Thank you so much for your comments. |
